# White matter changes following electroconvulsive therapy for depression: a mega-analysis

**DOI:** 10.1192/j.eurpsy.2022.237

**Published:** 2022-09-01

**Authors:** J.-B. Belge, P. Mulders, L. Van Diermen, A. Dols, M. Oudega, I. Tendolkar, P. Van Eijndhoven

**Affiliations:** 1 University of Antwerp, Collaborative Antwerp Psychiatric Research Institute (capri), Duffel, Belgium; 2 Radboud University Medical Center, Donders Institute For Brain, Cognition And Behavior, Centre For Neuroscience, Nijmegen, Netherlands; 3 Amsterdam University Medical Center, Amsterdam Neuroscience, Amsterdam, Netherlands

**Keywords:** Diffusion Tensor Imaging, Electroconvulsive therapy, Tract-Based Spatial Statistics, Depression

## Abstract

**Introduction:**

Electroconvulsive therapy (ECT) is proposed to exert an effect on white matter (WM) microstructure, but the limited power of previous studies made it difficult to highlight consistent patterns of change in diffusion metrics.

**Objectives:**

We initiated a multi-site mega-analysis and sought to address whether changes in WM microstructure occur following ECT.

**Methods:**

To this end, diffusion tensor imaging (DTI) data (n=58) from 4 different sites were harmonized before pooling them by using ComBat, a batch-effect correction tool that removes inter-site technical variability, preserves inter-site biological variability and maximizes statistical power. Downstream statistical analyses aimed to quantify changes in Fractional anisotropy (FA), Mean Diffusivity (MD), Radial Diffusivity (RD) and Axial Diffusivity (AD), by employing whole-brain, tract-based spatial statistics (TBSS).

**Results:**

ECT increases FA in the right splenium of the corpus callosum and the left cortico-spinal tract. Both the left superior longitudinal fasciculus and the right inferior fronto-occipital fasciculus showed increases in AD. Increases in MD and RD could be observed in overlapping white matter structures of both hemispheres. Finally, responders showed significantly smaller FA values in the left forceps major and smaller AD values in the right uncinate fasciculus compared with non-responders.

**Conclusions:**

This is the first and largest multi-site mega-analysis to demonstrate that ECT normalizes altered WM microstructure in important brain circuits that are implicated in the pathophysiology of depression. Furthermore, responders appear to present a more decreased WM integrity at baseline, which if replicated could serve as a biomarker for ECT response.

**Disclosure:**

No significant relationships.

